# A Perspective on Tongue Diagnosis in Patients with Breast Cancer

**DOI:** 10.1155/2021/4441192

**Published:** 2021-12-26

**Authors:** Po-Chi Hsu, Han-Kuei Wu, Hen-Hong Chang, Jia-Ming Chen, John Y. Chiang, Lun-Chien Lo

**Affiliations:** ^1^School of Chinese Medicine, China Medical University, Taichung, Taiwan; ^2^Department of Traditional Chinese Medicine, Kuang Tien General Hospital, Taichung, Taiwan; ^3^School of Post-Baccalaureate Chinese Medicine, China Medical University, Taichung, Taiwan; ^4^Department of Chinese Medicine, China Medical University Hospital, Taichung, Taiwan; ^5^Department of Chinese Medicine, Changhua Christian Hospital, Changhua, Taiwan; ^6^Department of Healthcare Administration and Medical Informatics, Kaohsiung Medical University, Kaohsiung, Taiwan; ^7^Department of Computer Science & Engineering, National Sun Yat-sen University, Kaohsiung, Taiwan

## Abstract

**Introduction:**

Breast cancer (BC) is the most common cancer in women and patients with BC often undergo complex treatment. In Taiwan, nearly 80% of patients with BC seek traditional Chinese medicine (TCM) during adjuvant chemotherapy to relieve discomfort and side effects. This study investigated tongue features and pattern differentiation through noninvasive TCM tongue diagnosis in patients with BC.

**Materials and Methods:**

This cross-sectional, case-controlled, retrospective observational study collected patient data through a chart review. The tongue features were extracted using the automatic tongue diagnosis system (ATDS). Nine tongue features, including tongue shape, tongue color, fur thickness, fur color, saliva, tongue fissures, ecchymoses, teeth marks, and red dots, were analyzed. *Results and Discussion*. Objective image analysis techniques were used to identify significant differences in the many tongue features between BC patients and non-BC individuals. A significantly larger proportion of patients with BC had a small tongue (*p* < 0.001), pale tongue (*p* < 0.001), thick fur (*p* < 0.001), yellow fur (*p* < 0.001), wet saliva (*p* < 0.001), thick tongue fur (*p* < 0.001), fissures (*p*=0.040), and ecchymoses in the heart-lung area (*p*=0.013). According to logistic regression, small tongue shape, pale tongue color, yellow fur color, wet saliva, and the amounts of fissures were associated with a significantly increased odds ratio for BC.

**Conclusions:**

This study showed significant differences in tongue features, such as small tongue shape, pale tongue color, thick fur, yellow fur color, wet saliva, fissure, and ecchymoses in the heart-lung area in patients with BC. These tongue features would imply yin deficiency, deficiencies of blood, stagnation of heat, and phlegm/blood stasis in TCM theory. There is a need to investigate effective and safe treatment to enhance the role of TCM in integrated medical care for patients with BC.

## 1. Background

Breast cancer (BC) is the most prevalent malignant tumor in women, accounting for 25.1% of all cancers [1]. The current treatment for patients with advanced BC includes surgery, radiotherapy, chemotherapy, and hormonal and targeted biological therapies [2]. Chemotherapy plays an important role in the systemic treatment of BC, and taxane-based or anthracycline-based chemotherapy may decrease cancer recurrence and mortality [3]. Currently, after breast surgery, the National Comprehensive Cancer Network (NCCN) guidelines specify only adjuvant chemotherapy to eliminate remaining cancer cells and improve survival of patients with early stages of BC [4]. However, chemotherapeutic agents can lead to discomfort and affect the quality of life (QOL), which influences the patient's compliance with treatment [5]. Recent studies have documented that at least 46% of patients with BC receive complementary and alternative medicine treatments to boost immune system activity, relieve discomfort, and increase QOL, and even as a cancer treatment [6, 7].

Traditional Chinese medicine (TCM) is provided as a complementary therapy to patients with BC and is an important component of healthcare in Taiwan. According to a study by Lai et al., approximately 81.5% of patients with BC had ever received complementary TCM treatment in Taiwan. [8] Another study has shown that adjunctive therapy with TCM may improve the survival of patients with advanced BC and receiving taxanes. [9] The available literature supports TCM's perceived effectiveness and its usage in the treatment of BC and BC-associated symptoms. [10, 11] However, TCM pattern differentiation in patients with BC is unknown. [12]

TCM physicians evaluate clinical symptoms and signs through four diagnostic methods, including inspection, listening, smelling, inquiry, and pulse feeling and palpation. Tongue diagnosis serves as a common noninvasive means to reliably provide clinical information and plays a pivotal role in TCM. By observing tongue features, TCM practitioners can determine the blood and qi status, and the alternation between yin and yang energies. These have a great impact on treatment and prognosis. [13, 14] Clinically, TCM practitioners observe tongue features, such as tongue color and shape, fur color and thickness, and the amount of saliva, to deduce the primary ailment of patients. The automatic tongue diagnosis system (ATDS) has shown high consistency and can provide objective and reliable information based on the analysis of tongue features, thereby assisting doctors in making effective observations and diagnoses of specific diseases. Several studies have used computerized tongue analysis to evaluate the relationship between tongue manifestations and various diseases, including rheumatoid arthritis, [15] BC, [16, 17] type 2 diabetes, [18, 19] metabolic syndrome, [20] eczema, [21] and dysmenorrhea. [22].

Patients with BC represent a unique group that requires complex and continuous care because several cancer treatment modalities can lead to side effects or complications, even after treatment completion. Understanding and interpreting these tongue manifestations in BC is important for both the theoretical and the clinical applications of TCM. This study applied ATDS to discriminate tongue features of patients with BC and without BC and provide valuable insights into accurate pattern differentiation and treatment.

## 2. Methods

### 2.1. Study Design and Data Sources

**Figure 1:**
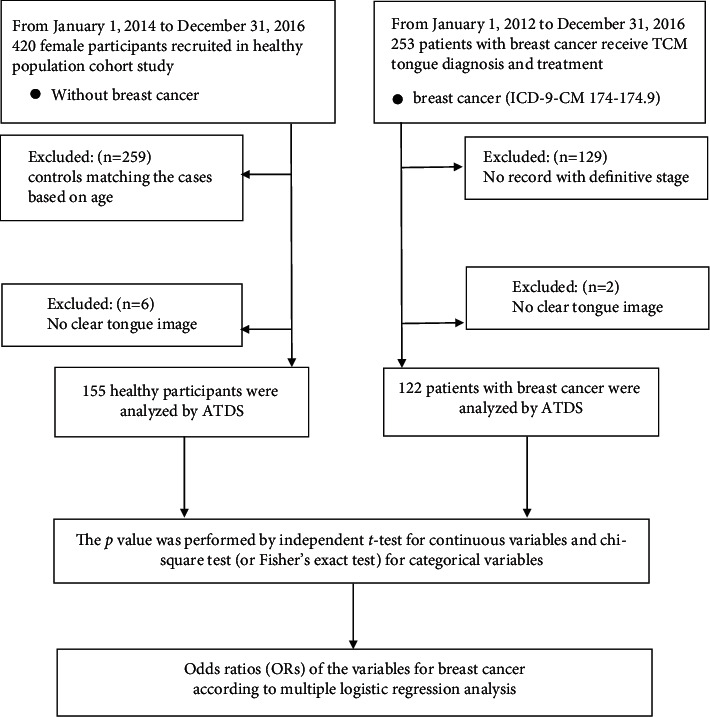
The exploratory flowchart for breast cancer-associated tongue diagnosis variates.

We conducted a retrospective case-controlled study from 2012 to 2016. We used the main data sources that included breast cancer and healthy population cohort study. In the breast cancer group, we first identified 253 patients with BC who received TCM tongue diagnosis and treatment in the TCM department of Changhua Christian Hospital (CCH), from January 1, 2012, to December 31, 2016. We excluded 129 subjects who did not completely record definitive stage, adjuvant chemotherapy regimens, and TCM treatment, and 2 subjects without clear tongue image. In the control group, we used the previous study and identified 420 female participants from January 1, 2012, to December 31, 2016. [23] We excluded 259 subjects due to matching of the cases based on age, and 6 subjects without clear tongue image. Finally, we enrolled 155 healthy participants and 122 patients with BC to undergo ATDS analysis (Figure 1). This study was approved by the Institutional Review Board (IRB) of CCH, Taiwan (IRB reference number: 111106 and 140704).

### 2.2. Inclusion Criteria

1. The current study comprised subjects aged >20 years who were diagnosed with primary breast cancer (ICD-9-CM 174–174.9) by a specialist
2. Only females were included

### 2.3. Exclusion Criteria

Subjects were excluded if they meet any of the following criteria:

1. Comorbidity of inadequate heart, liver, kidney, or other serious diseases
2. Pregnancy or lactation
3. History of mental illness

### 2.4. ATDS and Tongue Features

**Figure 2:**
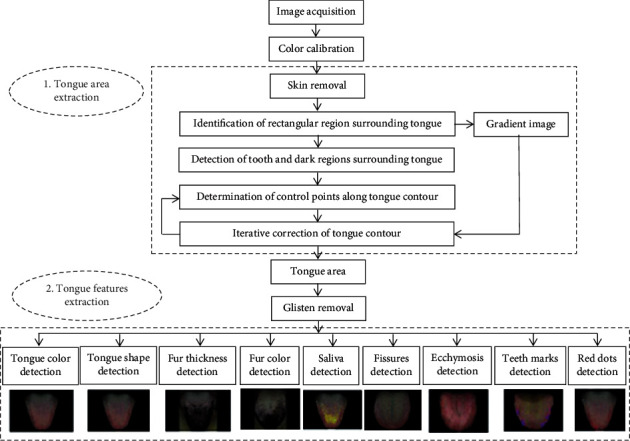
The processing flow of ATDS analysis.

**Figure 3:**
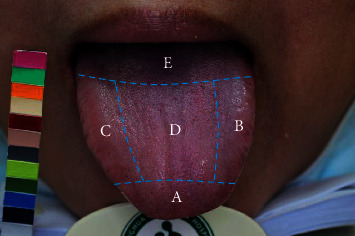
The tongue is subdivided into areas corresponding to different internal organs by ATDS. A: heart and lung area; B: left liver-gallbladder area; C: right liver-gallbladder area; D: spleen and stomach area; E: kidney area.

The ATDS was developed to capture tongue images and automatically extract features reliably to assist the diagnosis of TCM practitioners, as shown in Figure 2. The ATDS was developed by our team to capture tongue images and automatically extract features consistently to assist the diagnosis of TCM practitioners. The value of ATDS hinges on its ability to calibrate brightness and color to compensate for variations in intensity and color temperatures from light sources and imaging hardware. The tongue region is segmented automatically to derive relevant tongue features. There are nine primary features for TCM clinical tongue diagnosis, namely, tongue shape, tongue color, fur thickness, fur color, saliva, tongue fissure, ecchymosis, teeth mark, and red dot. Features identified are further subdivided according to the areas located (i.e., heart-lung area, left liver-gallbladder, right liver-gallbladder, spleen-stomach, and kidney) as shown in Figure 3.

A listing of the tongue features extracted is summarized as follows:

1. Tongue shape: including small, median, and enlarged
2. Tongue color: including pale, pink, red, and bluish
3. Fur thickness: degree of thickness (thin or thick), percentage of thick tongue fur (%)
4. Fur color: including white and yellow
5. Saliva: normal or wet
6. Tongue fissures: amount
7. Teeth marks: amount
8. Ecchymoses: amount and organs corresponding to the covering area (heart-lung, left liver-gallbladder, right liver-gallbladder, spleen-stomach, and kidney area)
9. Red dots: amount and organs corresponding to the covering area (heart-lung, left liver-gallbladder, right liver-gallbladder, spleen-stomach, and kidney area)

### 2.5. Data Analysis

The tongue features of the subjects who participated were extracted by ATDS and then compared between the BC group and control group to identify features with significant difference (*p* < 0.05). The data were expressed as mean ± standard deviation (SD), percentage, or number where appropriate. The tongue features in different groups were analyzed and compared using independent *t*-test for continuous variables and chi-square test (or Fisher's exact) for categorical variables. Logistic regression was used to estimate the odds ratio and the probability of a binary response based on one or more independent variables. Multiple logistic regression model for BC was performed by comparing with the participants without BC. Statistical analysis was performed using SPSS statistical software for Windows version 19 (SPSS for Windows, Version 19; SPSS Inc., Chicago, IL, USA). A *p* value <0.05 was considered statistically significant.

## 3. Results

The clinical characteristics of the control group and the patients with BC are shown in Table 1. A total of 155 participants in the control group and 122 patients with BC were enrolled in the study. The clinical characteristics of the participants were as follows: in the control group, average age was 52.7 ± 9.7 years and mean BMI 23.4 ± 3.3 kg/m^2^; and in BC group, average age was 51.5 ± 9.0 years and the mean BMI 23.5 ± 3.8 kg/m^2^. The patients with BC underwent current treatment strategies, such as surgery (55.7%), chemotherapy (31.1%), and radiation therapy (26.6%). The BC stages were classified as stage 0 (0.8%), stage I (26.2%), stage II (52.5%), stage III (17.2%), and stage IV (3.3%).

Tongue inspection refers to the examination of prominent tongue features, which include the body shape, tongue color, fur thickness, fur color, and saliva. Thus, the differences in tongue features between the BC and control groups were observed, as shown in Table 2. The small tongue shape was significantly more common in patients with BC than in the control group (32.8% vs. 12.3%, *p* < 0.001). The portion of patients with pale tongue color in the BC group was significantly higher than that in the control group (49.2% vs. 21.3%, *p* < 0.001). The portion of fur thickness in the BC group was significantly greater than that in the control group (48.4% vs. 21.6%, *p* < 0.001). Moreover, the proportion of yellow fur in the BC group was significantly greater than that in the control group (62.3% vs. 18.1%, *p* < 0.001). Furthermore, wet saliva was significantly common in patients with BC than that in the control group (42.6% vs.18.7%,*p* < 0.001). The differences in tongue characteristics between the BC and control groups were shown in Table 3. The percentage of thick tongue fur in the BC group was significantly higher than that in the control group (59.5% ± 23.0% vs. 46.3% ± 19.1%,*p* < 0.001). The mean numbers of fissures in the BC group were significantly higher than those in the control group (5.8 ± 7.0 vs. 4.2 ± 5.8, *p*=0.040), and the number of ecchymoses in the heart-lung area was significantly higher in the BC group than in the control group (4.8 ± 10.3 versus 2.5 ± 4.8, *p*=0.013). On the contrary, red dots were significantly less common in patients with BC than those in the control group (24.7 ± 23.4 vs. 67.8 ± 66.2, *p* < 0.001).

We identified the early stage (stages 0 and I) and advanced stage (stages II to IV) in patients with BC according to chart review and pathologic findings proved. The comparison of tongue features between early and advanced stages of BC was tabulated in Table 4. The proportion of wet saliva in the advanced stages of BC was less than that in the early stages (36.0% vs. 60.6%, *p*=0.014). Ecchymoses in the right liver-gallbladder area were significantly more common in the advanced stages of BC than in the early stages of BC (0.9 ± 2.2 vs. 0.3 ± 0.8, *p*=0.037). In contrast, the tongue features in tongue shape, tongue color, fur thickness, fur color, fissures, teeth marks, and red dots were not significantly different between the early and advanced stages of BC.

Logistic regression analysis by utilizing these 9 tongue features with significant differences was performed, shown in Table 5. In multiple logistic regression, small tongue shape (OR: 4.16, 95% CI:1.70–10.19, *p* < 0.001), pale tongue color (OR: 4.07, 95% CI: 1.84–8.99, *p* < 0.001), yellow fur color (OR: 6.06, 95% CI: 2.63–13.96, *p* < 0.001), wet saliva (OR: 4.19, 95% CI: 1.90–9.24, *p* < 0.001), and the number of fissures (OR: 1.08, 95% CI: 1.02–1.15, *p*=0.01) were associated with a significantly decreased risk of BC. On the other hand, the amounts of red dots (OR: 0.97, 95% CI: 0.96–0.98, *p* < 0.001) were associated with a significantly decreased risk of BC.

## 4. Discussion

This study aimed at exploring the tongue features and pattern differentiation through noninvasive TCM tongue diagnosis in patients with BC. Patients with BC represent a unique group that requires complex and continuous care because several cancer treatment modalities can lead to side effects or complications, even after treatment completion. An increasing number of patients are seeking TCM care to alleviate the side effects or complications resulting from cancer treatment. TCM has been used as an adjunct treatment for women with BC, as conventional treatments such as chemotherapy and radiation therapy can cause unpleasant side effects, including nausea, vomiting, fatigue, loose bowel movements, and hair loss. [24] There is a high prevalence of comorbidities and long-term complications, including cardiovascular diseases (CVD) secondary to treatment, bone diseases, second primary malignancies, lymphedema, thromboembolic events, and metabolic problems. [25] Several studies have investigated the use of TCM in cancer survivors and its effects on patient outcomes and medical costs. [26, 27] Some studies have suggested that patients with BC who integrated TCM with their conventional treatments experienced a higher survival rate, better tumor response during chemotherapy, lower all-cause mortality risk, and improved QOL. [9, 28, 29].

Positive therapeutic responses are dependent on proper pattern differentiation according to TCM theory and practice. Tongue diagnosis is a unique TCM method used to discriminate physiological functions and pathological conditions by observing the changes in the tongue features. [30] Tongue inspection refers to the visual evaluation of the shape, color, fur color, fur thickness, and other tongue characteristics. [31] According to the TCM treatment protocol for BC, there were several pattern differentiations, such as stagnation of liver-gi, spleen deficiency with phlegm dampness (plump tongue), deficiencies of liver and kidney yin (red tongue color with scanty fur), stagnation of heat and phlegm/stasis (red tongue with a yellow coat), and deficiencies of qi and blood (pale tongue). [12] Another study showed that the mirror-like tongue, thick tongue coating, and tongue moisture were more common in patients with cancers compared with healthy individuals. [32] Our study showed a significantly higher prevalence of specific tongue features, such as small tongue shape, pale tongue color, thick fur, yellow fur color, wet saliva, fissure, and ecchymoses in the heart-lung area in patients with BC compared with those in the control group. These tongue features implied pattern differentiations, such as yin deficiency, deficiencies of blood, stagnation of heat, and phlegm/stasis. Therefore, adaptive TCM treatment strategies, including nourishment of yin, blood, and removal of heat, dampness, phlegm, or stasis pathogens have been considered during cancer treatment. [33, 34] According to TCM theory and clinical experience, most patients with BC had more complex factors, including age, nutrition, comorbidity, and psychological and behavioral status. The relation of phlegm-stasis pathogens to the development of disease over time during treatment will need to be considered. The several differences in tongue features may contribute to a better understanding of the potential of TCM patterns for supplementing modern therapeutic strategies for BC.

The previous studies applied ATDS to investigate the tongue features to distinguish between patients with early stages of BC and individuals without BC. The TCM tongue diagnosis could serve as a preliminary screening procedure in early detection of BC in light of its simple and noninvasive way. [16, 17] Previous studies have shown that tongue ecchymosis is a characteristic sign of blood stasis. [18, 35] Furthermore, we compared tongue features between early and advanced stages of BC. Ecchymoses in the right liver-gallbladder area were significantly common in the advanced stages of BC than in the early stages of BC (0.9 ± 2.2 vs. 0.3 ± 0.8, *p*=0.037). We deduced that patients with BC went through several cancer treatment modalities that would lead to blood circulation disturbance. The study showed that the relation of phlegm-stasis syndrome and cancer-related fatigue in BC would need to be considered. [33] Continuous disturbances of the inner physical condition due to BC could lead to impaired blood circulation and cause blood stasis due to comorbidities and long-term complications. Our study showed that ecchymoses of the tongue were significantly higher in the heart-lung area and those in advanced stages were also significantly higher in right liver-gallbladder area. We proposed the reasons because pathological lesion of BC was located on the chest and the liver meridian. More studies are needed to determine whether tongue features are furtherly subdivided into areas (i.e., heart-lung area, left liver-gallbladder, right liver-gallbladder, spleen-stomach, and kidney area) corresponding to different internal organs. Understanding and interpreting these tongue manifestations in patients with BC is important in terms of theoretical and clinical applications. This study applied ATDS, a modern instrument with a high degree of consistency, to automatically evaluate the tongue features and, thus, reduce human vision bias. [16, 17].

However, there are several limitations to our study. First, because of data limitations, some clinical information, such as hormone receptor status and human epidermal growth factor receptor 2 status, and other variables like reproductive history and dietary habits, were not included in this study. Second, this study was a retrospective study and we selected patients with BC who visited the TCM department for complementary treatment. According to the previous studies, patients with BC integrated TCM with conventional medicine in strong connection, which could help improve the health outcomes. [36] We were not able to fully rule out the possibility of potential selection bias like group differences in patient's resources, health literacy, and care-seeking behaviors. Third, the evidence supporting the tongue's subdivisions and their correspondence to different internal organs is limited. Therefore, investigation with randomized controlled trials is required to validate these observational findings.

## 5. Conclusions

This study showed a significantly higher prevalence of tongue features, such as small tongue shape, pale tongue color, thick fur, yellow fur color, wet saliva, fissure, and ecchymoses in the heart-lung area in patients with BC. These tongue features implied pattern differentiation, such as yin deficiency, deficiencies of blood, stagnation of heat, and phlegm/blood stasis. Furthermore, there is a need to investigate effective and safe treatments to enhance the role of TCM in integrated medical care in patients with BC.

## Figures and Tables

**Table 1:** Demography of study participants.

Tongue features	Control	Breast cancer	*p* value
	*n* = 155	*n* = 122	
Age (mean ± SD)	52.7 ± 9.7	51.5 ± 9.0	0.338
BMI, kg/m^2^ (mean ± SD)	23.4 ± 3.3	23.5 ± 3.8	0.864
Surgery, *n* (%)		68 (55.7%)	
Chemotherapy regimens, *n* (%)		38 (31.1%)	
Radiation therapy, *n* (%)		32 (26.6%)	
Stage, *n* (%)			
Stage 0		1 (0.8%)	
Stage I		32 (26.2%)	
Stage II		64 (52.5%)	
Stage III		21 (17.2%)	
Stage IV		4 (3.3%)	

*p* values were performed by independent *t*-test for continuous variables. BMI: body mass index.

**Table 2:** Comparison of tongue features between patients with BC and control subjects.

Tongue features	Control	Breast cancer	*p* value
	*n* = 155	*n* = 122	
Tongue shape, *n* (%)			
Small	19 (12.3%)	40 (32.8%)	<0.001^*∗∗∗*^
Median	100 (64.5%)	68 (55.7%)	
Enlarged	36 (23.2%)	14 (11.5%)	
Tongue color, *n* (%)			
Pale	33 (21.3%)	60 (49.2%)	<0.001^*∗∗∗*^
Pink	89 (57.4%)	53 (43.4%)	
Red	24 (15.5%)	0 (0.0%)	
Bluish	9 (5.8%)	9 (7.4%)	
Fur thickness, *n* (%)			
Thin	122 (78.7%)	63 (51.6%)	<0.001^*∗∗∗*^
Thick	33 (21.3%)	59 (48.4%)	
Fur color, *n* (%)			
White	127 (81.9%)	46 (37.7%)	<0.001^*∗∗∗*^
Yellow	28 (18.1%)	76 (62.3%)	
Saliva, *n* (%)			
Normal	126 (81.3%)	70 (57.4%)	<0.001^*∗∗∗*^
Wet	29 (18.7%)	52 (42.6%)	

*p* values were performed by chi-square test (or Fisher's exact) for categorical variables. ^*∗*^*p* < 0.05, ^*∗∗*^*p* < 0.01, and ^*∗∗∗*^*p* < 0.001.

**Table 3:** Comparison of tongue characteristics between patients with BC and control subjects.

Tongue features	Control	Breast cancer	*p* value
	*n* = 155	*n* = 122	
Thick tongue fur (%)	46.3 ± 19.1	59.5 ± 23.0	<0.001^*∗∗∗*^
Fissures (mean ± SD)	4.2 ± 5.8	5.8 ± 7.0	0.040^*∗*^
Teeth marks (mean ± SD)	3.9 ± 2.6	3.4 ± 2.6	0.088
Ecchymoses (mean ± SD)	4.7 ± 8.2	6.7 ± 14.3	0.172
Ecchymoses in the heart-lung area	2.5 ± 4.8	4.8 ± 10.3	0.013^*∗*^
Ecchymoses in the left liver-gallbladder area	0.6 ± 1.3	0.5 ± 1.3	0.575
Ecchymoses in the right liver-gallbladder area	1.3 ± 2.9	0.7 ± 2.0	0.063
Ecchymoses in the spleen-stomach area	0.3 ± 1.3	0.7 ± 2.2	0.143
Ecchymoses in the kidney area	0 ± 0.1	0 ± 0.1	0.865
Red dots (mean ± SD)	67.8 ± 66.2	24.7 ± 23.4	<0.001^*∗∗∗*^
Red dots in the heart-lung area	31.4 ± 25.8	10.9 ± 12.6	<0.001^*∗∗∗*^
Red dots in the left liver-gallbladder area	9.2 ± 12.1	4.8 ± 6.2	<0.001^*∗∗∗*^
Red dots in the right liver-gallbladder area	17.0 ± 24.4	6.4 ± 7.2	<0.001^*∗∗∗*^
Red dots in the spleen-stomach area	8.4 ± 17.3	2.0 ± 5.2	<0.001^*∗∗∗*^
Red dots in the kidney area	1.8 ± 3.7	0.6 ± 1.4	<0.001^*∗∗∗*^

*p* values were performed by independent *t*-test for continuous variables. ^*∗*^*p* < 0.05, ^*∗∗*^*p* < 0.01, and ^*∗∗∗*^*p* < 0.001.

**Table 4:** Comparison of tongue features between early and advanced stages of BC.

Tongue features	Early stages of BC (stages 0 & I)	Advanced stages of BC (stages II& III & IV)	*p* value
	*n* = 33	*n* = 89	
Tongue shape, *n* (%)			
Small	11 (33.3%)	29 (32.6%)	0.332
Median	16 (48.5%)	52 (58.4%)	
Enlarged	6 (18.2%)	8 (9.0%)	
Tongue color, *n* (%)			
Pale	20 (60.6%)	40 (44.9%)	0.204
Pink-red	10 (30.3%)	43 (48.3%)	
Bluish	3 (9.1%)	6 (6.7%)	
Fur thickness, *n* (%)			
Thin	19 (57.6%)	44 (49.4%)	0.342
Thick	14 (42.4%)	45 (50.6%)	
Fur color, *n* (%)			
White	12 (36.4%)	34 (38.2%)	0.852
Yellow	21 (63.6%)	55 (61.8%)	
Saliva, *n* (%)			
Normal	13 (39.4%)	57 (64.0%)	0.014^*∗*^
Wet	20 (60.6%)	34 (36.0%)	
Thick tongue fur	60.7 ± 23.1	59.1 ± 23.1	0.730
Fissures (mean ± SD)	5.0 ± 6.2	6.1 ± 7.3	0.439
Teeth marks (mean ± SD)	3.2 ± 2.9	3.5 ± 2.6	0.606
Ecchymoses (mean ± SD)	6.8 ± 12.9	6.7 ± 14.8	0.986
Ecchymoses in the heart-lung area	5.5 ± 10.9	4.5 ± 10.2	0.642
Ecchymoses in the left liver-gallbladder area	0.4 ± 0.9	0.6 ± 1.4	0.509
Ecchymoses in the right liver-gallbladder area	0.3 ± 0.8	0.9 ± 2.2	0.037^*∗*^
Ecchymoses in the spleen-stomach area	0.5 ± 1.5	0.7 ± 2.5	0.723
Ecchymoses in the kidney area	0	0 ± 0.1	0.545
Red dots (mean ± SD)	26.1 ± 22.3	24.1 ± 23.9	0.686
Red dots in the heart-lung area	10.6 ± 12.5	11.0 ± 12.7	0.892
Red dots in the left liver-gallbladder area	5.3 ± 6.4	4.6 ± 6.2	0.563
Red dots in the right liver-gallbladder area	7.6 ± 8.5	6.0 ± 6.7	0.276
Red dots in the spleen-stomach area	2.1 ± 4.5	2.0 ± 5.5	0.938
Red dots in the kidney area	0.5 ± 1.0	0.6 ± 1.6	0.630

*p* values were performed by independent *t*-test for continuous variables and chi-square test (or Fisher's exact) for categorical variables. ^*∗*^*p* < 0.05.

**Table 5:** Odds ratios (ORs) of the tongue features for BC according to multiple logistic regression.

	Odds ratio	95% exp (B)		*p* value^a^
		Lower	Upper	
Tongue shape (median)				
Small	4.16	1.70	10.19	<0.001^*∗∗∗*^
Enlarged	0.48	0.18	1.30	0.15
Tongue color (pink)				
Pale	4.07	1.84	8.99	<0.001^*∗∗∗*^
Red	0.00	0.00	—	0.99
Bluish	1.83	0.49	6.80	0.37
Fur thickness	1.65	0.48	5.77	0.43
Yellow fur color	6.06	2.63	13.96	<0.001^*∗∗∗*^
Wet saliva	4.19	1.90	9.24	<0.001^*∗∗∗*^
Thick tongue fur	0.99	0.97	1.02	0.77
The amounts of fissures	1.08	1.02	1.15	0.01^*∗*^
The amounts of ecchymoses	1.02	0.98	1.05	0.35
The amounts of red dots	0.97	0.96	0.98	<0.001^*∗∗∗*^

^a^Multiple logistic regression model for breast cancer was performed by comparing with the participants without BC. ^*∗*^*p* < 0.05, ^*∗∗*^*p* < 0.01, and ^*∗∗∗*^*p* < 0.001.

## Data Availability

The datasets generated and/or analyzed during the current study are not publicly available but are available from the corresponding author on reasonable request.

## References

[1] Ghoncheh M., Pournamdar Z., Salehiniya H. (2016). Incidence and mortality and epidemiology of breast cancer in the world. *Asian Pacific Journal of Cancer Prevention*.

[2] Veronesi U., Boyle P., Goldhirsch A., Orecchia R., Viale G. (2005). Breast cancer. *Lancet*.

[3] Early Breast Cancer Trialists’ Collaborative Group (2012). Comparisons between different polychemotherapy regimens for early breast cancer: meta-analyses of long-term outcome among 100,000 women in 123 randomised trials. *The Lancet*.

[4] Mackey J. R., Martin M., Pienkowski T. (2013). Adjuvant docetaxel, doxorubicin, and cyclophosphamide in node-positive breast cancer: 10-year follow-up of the phase 3 randomised BCIRG 001 trial. *The Lancet Oncology*.

[5] Calderon C., Carmona-Bayonas A., Hernández R. (2019). Effects of pessimism, depression, fatigue, and pain on functional health-related quality of life in patients with resected non-advanced breast cancer. *The Breast*.

[6] Saquib J., Madlensky L., Kealey S. (2011). Classification of CAM use and its correlates in patients with early-stage breast cancer. *Integrative Cancer Therapies*.

[7] Templeton A. J., Thürlimann B., Baumann M. (2013). Cross-sectional study of self-reported physical activity, eating habits and use of complementary medicine in breast cancer survivors. *BMC Cancer*.

[8] Lai J.-N., Wu C.-T., Wang J.-D. (2012). Prescription pattern of Chinese herbal products for breast cancer in taiwan: a population-based study. *Evidence-based Complementary and Alternative Medicine*.

[9] Lee Y.-W., Chen T.-L., Shih Y.-R. V. (2014). Adjunctive traditional Chinese medicine therapy improves survival in patients with advanced breast cancer: a population-based study. *Cancer*.

[10] Chen Z., Gu K., Zheng Y., Zheng W., Lu W., Shu X. O. (2008). The use of complementary and alternative medicine among Chinese women with breast cancer. *Journal of Alternative & Complementary Medicine*.

[11] McPherson L., Cochrane S., Zhu X. (2016). Current usage of traditional Chinese medicine in the management of breast cancer: a practitioner’s perspective. *Integrative Cancer Therapies*.

[12] Liu C.-T., Chen Y.-H., Huang Y.-C., Chen S.-Y., Tsai M.-Y. (2019). Chemotherapy in conjunction with traditional Chinese medicine for survival of patients with early female breast cancer: protocol for a non-randomized, single center prospective cohort study. *Trials*.

[13] Kirschbaum B. (2000). *Atlas of Chinese Tongue Diagnosis*.

[14] Anastasi J. K., Currie L. M., Kim G. H. (2009). Understanding diagnostic reasoning in TCM practice: tongue diagnosis. *Alternative Therapies in Health and Medicine*.

[15] Lo L., Chen C., Chiang J., Cheng T., Lin H., Chang H. (2013). Tongue diagnosis of traditional Chinese medicine for rheumatoid arthritis. *African Journal of Traditional, Complementary and Alternative Medicines*.

[16] Lo L.-c., Cheng T.-L., Chen Y.-J., Natsagdorj S., Chiang J. Y. (2015). TCM tongue diagnosis index of early-stage breast cancer. *Complementary Therapies in Medicine*.

[17] Lo L.-C., Cheng T.-L., Chiang J. Y., Damdinsuren N. (2013). Breast cancer index: a perspective on tongue diagnosis in traditional Chinese medicine. *Journal of Traditional and Complementary Medicine*.

[18] Hsu P.-C., Huang Y.-C., Chiang J. Y., Chang H.-H., Liao P.-Y., Lo L.-C. (2016). The association between arterial stiffness and tongue manifestations of blood stasis in patients with type 2 diabetes. *BMC Complementary and Alternative Medicine*.

[19] Hsu P.-C., Wu H.-K., Huang Y.-C. (2019). The tongue features associated with type 2 diabetes mellitus. *Medicine (Baltimore)*.

[20] Lee T.-C., Lo L.-C., Wu F.-C. (2016). Traditional Chinese medicine for metabolic syndrome via TCM pattern differentiation: tongue diagnosis for predictor. *Evidence-based Complementary and Alternative Medicine*.

[21] Yu Z., Zhang H., Fu L., Lu X. (2017). Objective research on tongue manifestation of patients with eczema. *Technology and Health Care*.

[22] Kim J., Lee H., Kim H., Kim J. Y., Kim K. H. (2017). Differences in the tongue features of primary dysmenorrhea patients and controls over a normal menstrual cycle. *Evidence-based Complementary and Alternative Medicine*.

[23] Hsu P.-C., Wu H.-K., Huang Y.-C. (2019). Gender- and age-dependent tongue features in a community-based population. *Medicine (Baltimore)*.

[24] Cui Y., Shu X.-O., Gao Y. (2004). Use of complementary and alternative medicine by Chinese women with breast cancer. *Breast Cancer Research and Treatment*.

[25] Bodai B., Tuso P. (2015). Breast cancer survivorship: a comprehensive review of long-term medical issues and lifestyle recommendations. *The Permanente Journal*.

[26] Lu S.-Y., Chen J.-J., Pan J.-I., Fu Z.-X., Wu J.-L., Hsieh T.-C. (2019). The association between different patterns of traditional Chinese medicine treatment and all-cause mortality among cancer patients. *Integrative Cancer Therapies*.

[27] Sun X., Zhang X., Nian J.-Y. (2016). Chinese herbal medicine as adjunctive therapy to chemotherapy for breast cancer: a systematic review and meta-analysis. *Evidence-based Complementary and Alternative Medicine*.

[28] Porter D., Cochrane S., Zhu X. (2017). Current usage of traditional Chinese medicine for breast cancer-A narrative approach to the experiences of women with breast cancer in Australia-A pilot study. *Medicine*.

[29] Kuo Y.-T., Chang T.-T., Muo C.-H. (2018). Use of complementary traditional Chinese medicines by adult cancer patients in taiwan: a nationwide population-based study. *Integrative Cancer Therapies*.

[30] Anastasi J. K., Currie L. M., Kim G. H. (2009). Understanding diagnostic reasoning in TCM practice: tongue diagnosis. *Alternative Therapies in Health and Medicine*.

[31] Lo L.-C., Chiang J. Y., Cheng T.-L., Shieh P.-S. (2012). Visual agreement analyses of traditional Chinese medicine: a multiple-dimensional scaling approach. *Evidence-based Complementary and Alternative Medicine*.

[32] Han S., Yang X., Qi Q. (2016). Potential screening and early diagnosis method for cancer: tongue diagnosis. *International Journal of Oncology*.

[33] Deng S.-M., Chiu A.-F., Wu S.-C. (2021). Association between cancer-related fatigue and traditional Chinese medicine body constitution in female patients with breast cancer. *Journal of Traditional and Complementary Medicine*.

[34] Chen R., Moriya J., Yamakawa J.-i., Takahashi T., Kanda T. (2010). Traditional Chinese medicine for chronic fatigue syndrome. *Evidence-based Complementary and Alternative Medicine*.

[35] Ren Q., Zhou X.-w., He M.-y. (2020). A quantitative diagnostic method for phlegm and blood stasis syndrome in coronary heart disease using tongue, face, and pulse indexes: an exploratory pilot study. *Journal of Alternative & Complementary Medicine*.

[36] Yeh C.-M., Chou Y.-J., Lin S.-K., Liu C.-J., Huang N. (2021). Patient-sharing relationship between Chinese medicine doctors and other physicians: costs and outcomes of breast cancer survivorship care. *Journal of Cancer Survivorship*.

